# Mathematical Modelling of a Brain Tumour Initiation and Early Development: A Coupled Model of Glioblastoma Growth, Pre-Existing Vessel Co-Option, Angiogenesis and Blood Perfusion

**DOI:** 10.1371/journal.pone.0150296

**Published:** 2016-03-02

**Authors:** Yan Cai, Jie Wu, Zhiyong Li, Quan Long

**Affiliations:** 1 State Key Laboratory of Bioelectronics, Southeast University, Nanjing, China; 2 School of Biological Science and Medical Engineering, Southeast University, Nanjing, China; 3 School of Naval Architecture, Ocean and Civil Engineering, Shanghai Jiaotong University, Shanghai, China; 4 Brunel Institute for Bioengineering, School of Engineering and Design, Brunel University, Uxbridge, Middlesex, United Kingdom; University of Michigan School of Medicine, UNITED STATES

## Abstract

We propose a coupled mathematical modelling system to investigate glioblastoma growth in response to dynamic changes in chemical and haemodynamic microenvironments caused by pre-existing vessel co-option, remodelling, collapse and angiogenesis. A typical tree-like architecture network with different orders for vessel diameter is designed to model pre-existing vasculature in host tissue. The chemical substances including oxygen, vascular endothelial growth factor, extra-cellular matrix and matrix degradation enzymes are calculated based on the haemodynamic environment which is obtained by coupled modelling of intravascular blood flow with interstitial fluid flow. The haemodynamic changes, including vessel diameter and permeability, are introduced to reflect a series of pathological characteristics of abnormal tumour vessels including vessel dilation, leakage, angiogenesis, regression and collapse. Migrating cells are included as a new phenotype to describe the migration behaviour of malignant tumour cells. The simulation focuses on the avascular phase of tumour development and stops at an early phase of angiogenesis. The model is able to demonstrate the main features of glioblastoma growth in this phase such as the formation of pseudopalisades, cell migration along the host vessels, the pre-existing vasculature co-option, angiogenesis and remodelling. The model also enables us to examine the influence of initial conditions and local environment on the early phase of glioblastoma growth.

## Introduction

Gliomas are the most common central nervous system tumours and carry high rates of morbidity and mortality. The 4-level grading system proposed by the World Health Organization (WHO) in 1993 was widely accepted and widespread [1]. As the most malignant and also the most frequent gliomas, Grade IV tumours including glioblastoma (GBM) and gliosarcoma, can develop from a lower grade tumour, metastasize from other tumours or directly from glioblastoma cells and have a mortality rate close to 100%. Due to its special growth pattern where tumour cells normally surround and attach to microvessel walls, surgical operation cannot remove the tumour efficiently. Non-surgical therapies have much more important roles in brain tumour treatment. A good understanding of the interactions between GBM growth and tumour microenvironment will be necessary for the design and evaluation of anti-tumour therapy [2].

An important pathological feature that distinguishes GBM from lower-grade brain tumours is the necrotic foci which are typically surrounded by hypercellular zones referred to as pseudopalisades [3]. Brat *et al* [4] revealed that pseudopalisades in GBM are hypoxic, express extracellular matrix degradation enzymes (MDEs), and are formed by an actively migrating cell population. They proposed two potential mechanisms of pseudopalisade formation: (a) tumour cells at greatest distance from arterial supplies become hypoxic due to increased metabolic demands, leaving a central necrotic core; (b) microvascular occlusion or collapse within the neoplasm could lead to perivascular hypoxia. As straightforward evidence of the latter, many pseudopalisades have a long, narrow, and winding pattern, which suggests an underlying vascular substrate associated with their emergence.

Pseudopalisades are pathophysiologically linked with adjacent microvascular hyperplasia, which can express high levels of angiogenic regulators and inducing tumour angiogenesis [5]. In addition, the "co-option" of pre-existing vessel networks plays a significant role in glioma progression. Holash *et al* [6] showed that even the smallest C6 gliomas at just 1 week after implantation were well vascularized by co-option of pre-existing blood vessels. Further experiments [7–9] revealed that when a small number of tumour cells were implanted into healthy tissue, they managed to co-opt and migrate along host vessels, produce many chemical substances, such as vascular endothelial growth factors (VEGFs), Ang-1, Ang-2, to change the microenvironment around the host vessels. These can induce immature changes in the host tissue vasculature, including vessel dilation, increased capillary permeability and tortuosity [10]. With tumour growth, cancer cells migrate along the blood vessels, compressing and destabilizing them, which leads to vessel regression and reduced blood perfusion [11,12].

Mathematical modelling has been used for the study of the interaction between tumour growth and local microenvironment for many years. The early stage models were generally of the simulation of single phenomena and static states, such as tumour angiogenesis [13] and tumour growth under the influence of local stress (pressure) [14]. The dynamic interactions among a few phenomenon such as angiogenesis and blood flow perfusion, without [15–19] or with the coupling of an interstitial fluid field [20–23] are the main focuses in recent works. Tumour cell evolution was studied by superimposing local microenvironments such as oxygen distributions, or coupling with local vasculatures [24–27]. With the development of model complexity, more pathophysiological characteristics can be coupled in a model which allows us to study the dynamic interactions among the phenomenan. The simulation results will also be more realistic with fewer superimposed assumptions. For example, by the inclusion of pre-existing microvessels in tumour cell sites, models with vessel regression and vessel co-option can be used to study early phase tumour development and the dynamics of tumour growth with local initial conditions [28,29].

Although mathematical modelling contributes greatly in improving our understanding of generic solid tumour development and its interactions with local microenvironments [30], studies specifically on brain tumours are still rare [31–33]. Stein *et al* [34] developed a continuum mathematical model of the dispersion behaviours of glioblastoma tumour spheroid with the aim of identifying and characterizing discrete cellular mechanisms underlying invasive cell motility. Swanson *et al* [35–39] did a series of outstanding work on *in silico* modelling of glioma proliferation and invasion kinetics Their models were based on the classic conservation equation of tumour cell population, in which the active motility of glioma cells was assumed to satisfy the gradient-driven Fickian diffusion. Their recent study [39] proposed the Proliferation, Invasion, Hypoxia, Necrosis, Angiogenesis (PIHNA) model by incorporating the angiogenic cascade-based net rates and concentrations of cell populations which interact, proliferate, decay, and migrate. This model provided a fairly systematic description of interaction of gliomas with its microenvironment. However, there is no haemodynamic calculation in their models. The pseudopalisades structure was not mentioned in their study. Martínez-González *et al* [40] presented a mathematical model to describe pseudopalisades in GBM as hypoxic cell waves around necrotic cores. The model consisted of only two parallel blood vessels and an evolving embedded population of tumour cells whose two phenotypes changed according to the oxygen level. Their simulation results revealed the formation of a traveling wave of hypoxic cells that qualitatively reproduced the experimentally observed patterns. In addition, mathematical modelling has been introduced to study the treatment of brain tumours, such as tumour resection [41], chemotherapy [42] and anti-angiogenic therapies [43]. However, certain important pathophysiological characteristics in malignant progression of GBM, especially the dynamic process of pre-existing vasculature remodelling and its influences on tumour growth, are neglected in their published studies.

The present study aims to develop a mathematical model which is capable of simulating the dynamic processes of tumour cell proliferation, migration, co-option of pre-existing vessels and angiogenesis, coupled with blood perfusion at the early stage of GBM growth. A 3D tree-like architecture network with different orders for vessel diameter is generated as pre-existing vasculature in host tissue. The chemical substances including oxygen, vascular endothelial growth factor, extra-cellular matrix and matrix degradation enzymes are calculated based on the haemodynamic environment which is obtained by coupled modelling of intravascular blood flow with interstitial fluid flow. The haemodynamic changes, including vessel diameter and permeability, are introduced to reflect a series of pathological characteristics of abnormal tumour vessels including vessel dilation, leakage, angiogenesis, regression and collapse. The model adopted the following assumptions based on the corresponding experimental and clinical observations: (a) the migration speed of small groups of cancer cells along the host vessel longitudinal direction is faster than in a radial direction from the vessel, (b) vessel maturation is estimated by the vessel dilation and the increased wall permeability. With all of these newly added coupled simulation, as part of validation, we like to use this paper to reproduce the observed pathophysiological phenomenan of GBM such as (i) glioma cell migration along the host vessel, (ii) formation of pseudopalisades, (iii) pre-existing vessel co-option, remodelling and collapse. In addition, the influence of the ability of glioma cell migration, the inclusion of vessel immature status due to GBM growth and their contributions to the formation of pseudopalisades will be presented and discussed in the subsequent sections.

## Method

### Pre-existing vessel network

**Fig 1:**
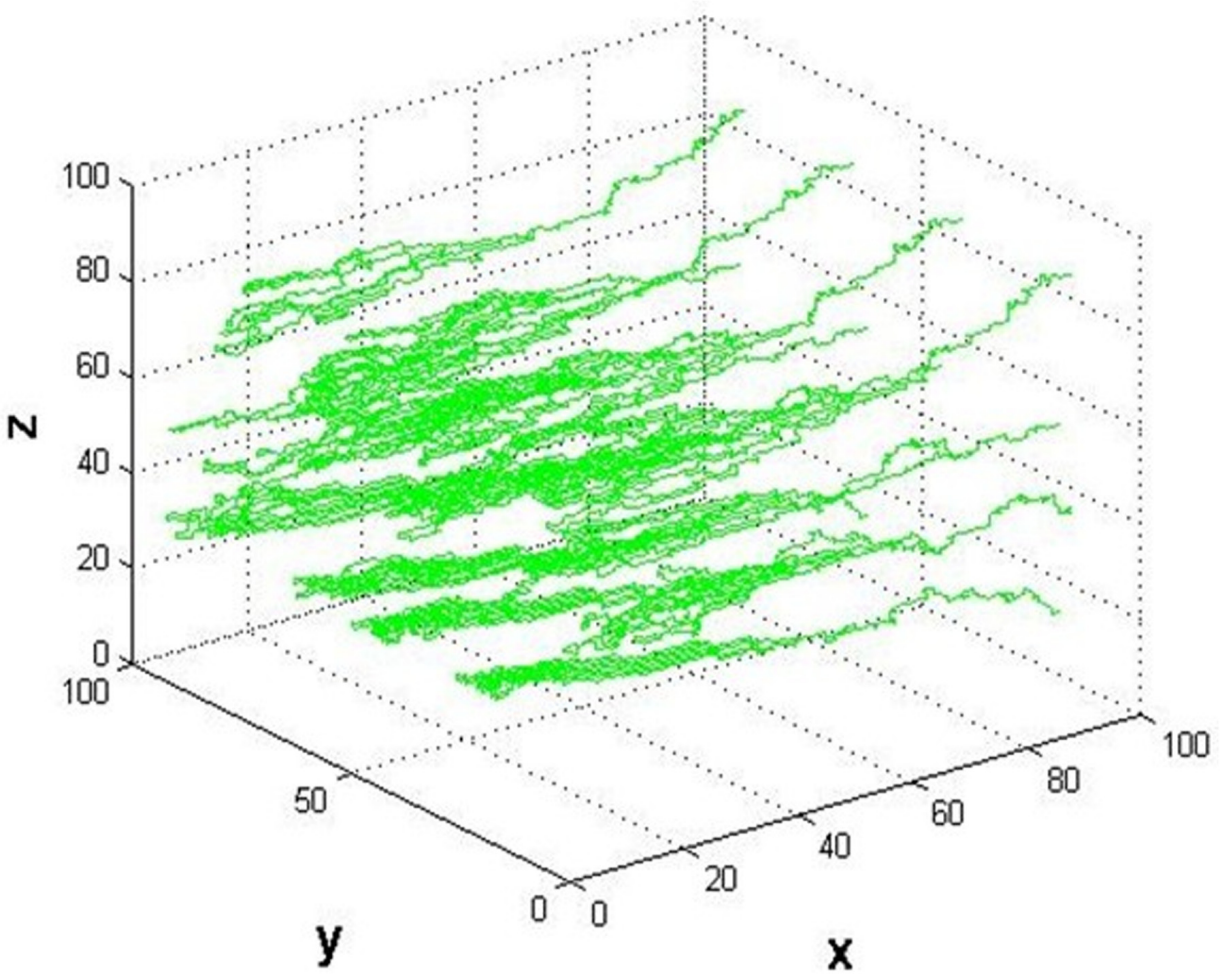
Pre-existing microvessel network. The typical tree-like architecture network of pre-existing vessels in the basic model.

**Fig 2:**
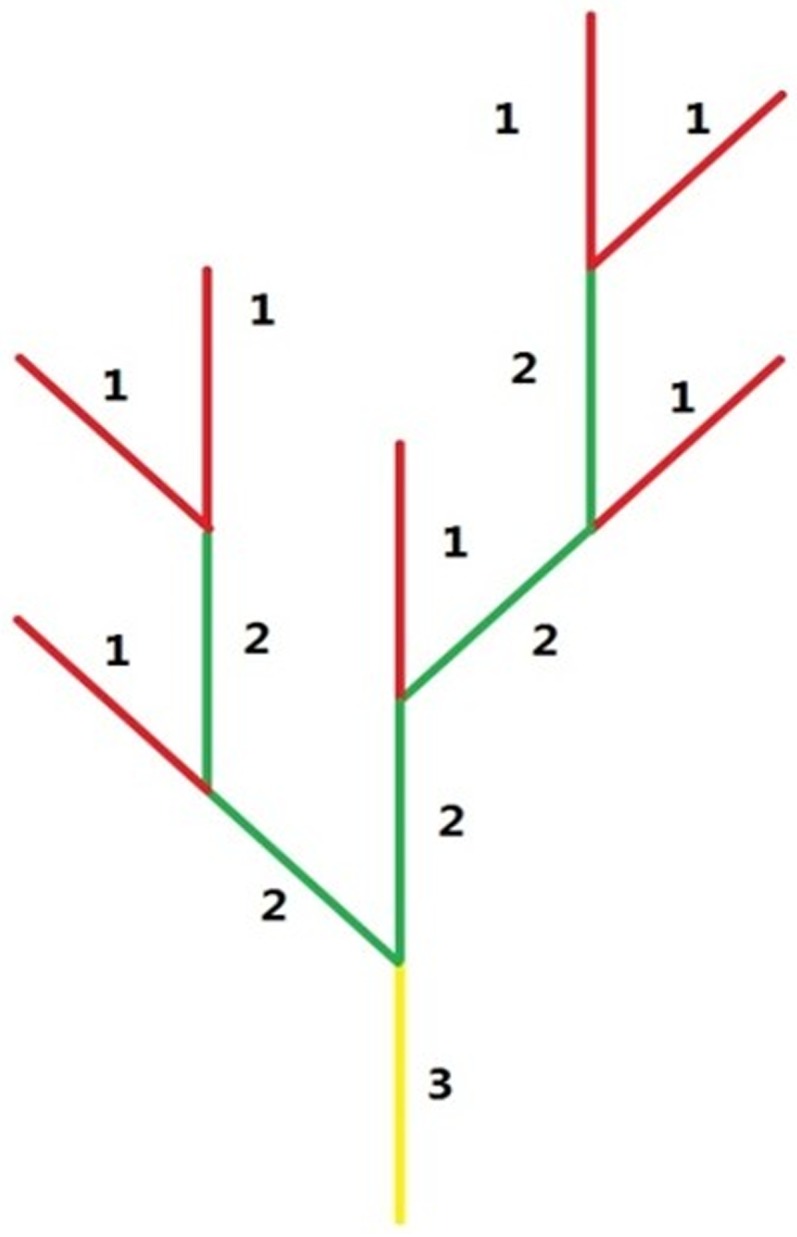
The Strahler system for pre-existing vasculature.

For the morphological analysis we consider vessel segments within a cube simulation domain Ω of 1mm^3^. A basic grid of 100x100x100 is generated uniformly in the cube with a centre to centre length of 10μm between the neighbouring nodes (Fig 1). The pre-existing vasculature in the basic model has a typical tree-like architecture network in accord with the features of human cerebral microcirculation in Cassot *et al*’s experimental work [44]. For the topological analysis, we classify vessel branches according to the Strahler system [44], a well-established method for describing stream order (Fig 2). The vessel diameter was defined according to the vessel Strahler order. In Strahler’s system, leaf segments are assigned Strahler order one. The Strahler order will increase when two vessels with the same Strahler orders join into one vessel. However, two vessels with different Strahler order meeting will not create a vessel with higher order. In our model, there are three Strahler orders to show a brief tree architecture of an arteriolar branching pattern. The main stem of trees, with a Strahler order 3, grow approximately in vertical direction from plane x = 100 to x = 0, and have the biggest value of vessel diameter. Mean values of capillary diameter and length respectively of different orders of vascular tree are shown in Table 1, based on the measurements by Cassot *et al* [44].

**Table 1:** Initial values of microvessels with different Strahler orders.

Strahler order	1	2	3
Diameter (μm) [26]	8	12	16
Length (μm) [26]	80	200	320
Pc (mmHg) [33]	1.0	1.5	2.0

The diameter and average length, and the collapse pressure of vessel segments of different Strahler orders.

### Haemodynamic calculation

A number of numerical models have been developed in recent years on brain blood flow in an anatomically accurate human cerebral vascular network [45–48]. However, the models were generally designed to study blood perfusion in the brain under normal physiological conditions without the coupled effect of interstitial fluid flow and transvascular flow across the capillary network. The haemodynamic model in this study is based on our previous work on the coupled modelling of intravascular blood flow with interstitial fluid flow [20,21]. Briefly, the basic equation for the intravascular blood flow is the flux concentration and incompressible flow at each node. Poiseuille's law, Darcy's law and Starling's law were used to govern intra-vessel flow resistance, interstitial flow and trans-vessel flow respectively. Blood viscosity is a function of vessel diameter, local haematocrit, and plasma viscosity [49]. In addition, vessel compliance and wall shear stress are correlated to vessel remodelling and vessel collapse, which will be explained in details in the following section.

The main equations for blood flow calculation are as follows:

$${\text{Q}}_{\text{v}}=\frac{{\pi \text{R}}^{4}\Delta {\text{P}}_{\text{v}}}{8\mu \Delta \text{l}}$$ (1)

$${\text{Q}}_{\text{t}}=2\pi \text{R}\cdot \Delta \text{l}\cdot {\text{L}}_{\text{p}}\left({\text{P}}_{\text{v}}-{\text{P}}_{\text{i}}-\sigma_{\text{T}}\left(\pi_{\text{v}}-\pi_{\text{i}}\right)\right)$$ (2)

$$\text{Q}={\text{Q}}_{\text{v}}-{\text{Q}}_{\text{t}}$$ (3)

where Q is the flow rate of each vessel segment, which has a value zero at each node of the vessel network due to the assumption of flux conservation and incompressible flow. Q_v_ is the vascular flow rate without fluid leakage; Q_t_ is the transvascular flow rate. Δl and R are the mean length and radius of the vessel segment. Pv and Pi are the intravascular pressure and the interstitial pressure, respectively. Lp is the hydraulic permeability of the vessel wall. σ_T_is the average osmotic reflection coefficient for plasma proteins; π_v_ and π_i_ are the colloid osmotic pressure of plasma and interstitial fluid, respectively. The total difference of Pv from plane x = 100 to x = 0 is set to be 3.5mmHgas the driving force of blood in the network (or the boundary condition).

The velocity of intravascular Uv and interstitial flow Ui satisfies

$${\text{U}}_{\text{v}}=\text{Q}/{\pi \text{R}}^{2}$$ (4)

$${\text{U}}_{\text{i}}=-\text{K}\nabla {\text{P}}_{\text{i}}$$ (5)

$$\nabla \cdot {\text{U}}_{\text{i}}=\frac{{\text{L}}_{\text{p}}\text{S}}{\text{V}}\left({\text{P}}_{\text{v}}-{\text{P}}_{\text{i}}-\sigma_{\text{T}}\left(\pi_{\text{v}}-\pi_{\text{i}}\right)\right)$$ (6)

where K is the hydraulic conductivity coefficient of the interstitium; S/V is the surface area per unit volume for transport in the interstitium.

The distribution of red blood cells (RBCs) at a microvascular bifurcation is calculated based on the approach proposed by Pries [49]. The details of blood rheology simulation were described in Wu *et al* [20].

From the haemodynamic simulation, we are able to obtain (a) intravascular flow velocity Uv and the haematocrit H in the microvessel network which are used in the oxygen concentration calculation, and (b) vessel diameter which is used to estimate vessel remodelling and collapse.

### Chemicals concentration calculation

The glioma cell and endothelial cell behaviours are coupled by the changes of the chemicals in the extra-cellular matrix (ECM), such as oxygen, VEGF and MDEs. The transport of these chemicals (oxygen, VEGF and MDEs) are modelled by quasi-steady reaction-diffusion equations. The ECM is treated as a continuous substance and can be degraded by MDEs The MDEs are produced by TCs and ECs and the decay of itself which are all governed by diffusion. To obtain a more realistic oxygen concentration field, the advection and diffusion of oxygen in the vessel network are introduced, based on the work of Fang *et al* [50]. The computational space for oxygen calculation is separated into three domains to characterize three distinct physiological processes, which are (a) the oxygen advection equation inside the vessel, (b) the oxygen flux across the vessel wall and (c) the free oxygen diffusion in the interstitial tissue.

The equations describing the interactions of TCs and ECs with ECM and MDE are

$$\frac{\partial {\text{C}}_{\text{f}}}{\partial \text{t}}=-{\delta \text{C}}_{\text{m}}{\text{C}}_{\text{f}}$$ (7)

$$\frac{\partial {\text{C}}_{\text{m}}}{\partial \text{t}}={\text{D}}_{\text{m}}\nabla^{2}{\text{C}}_{\text{m}}+\mu_{\text{T}}{\text{TC}}_{\text{i},\text{j}}+\mu_{\text{E}}{\text{EC}}_{\text{i},\text{j}}-{\lambda \text{C}}_{\text{m}}$$ (8)

where C_f_ and C_m_ are the ECM and MDE concentration, separately. The TC_i,j_ and EC_i,j_ terms represent a tumour cell and an endothelial cell located at a node position (i,j). Their values are either 1 if a cell is present or 0 if it is not. D_m_ is the MDE diffusion coefficient, and, δ, μ_T_, μ_E,_ λ are positive constants.

VEGF is assumed to diffuse, decay and be consumed by angiogenic sprouts. The production of VEGF is assumed to be proportional to TCs and ECM, representing the secretion of VEGF by TCs and the up-regulated level of VEGF in the ECM. The equation of VEGF concentration C_v_ is as follows

$$\frac{\partial {\text{C}}_{\text{v}}}{\partial \text{t}}={\text{D}}_{\text{v}}\nabla^{2}{\text{C}}_{\text{v}}+{\chi \text{TC}}_{\text{i},\text{j}}+{\xi \text{C}}_{\text{f}}-{\varepsilon \text{EC}}_{\text{i},\text{j}}-{\theta \text{C}}_{\text{v}}$$ (9)

where D_v_ is VEGF diffusion coefficient, and, χ, ξ, ε, θ are positive constants.

The oxygen transport inside the vessel is represented by the advection equation subject to the equilibrium of the free and bound oxygen:

$$\frac{\partial {\text{C}}_{\text{o}\_\text{F}}^{\text{in}}}{\partial \text{t}}=-\vec{{\text{U}}_{\text{v}}}\cdot \nabla {\text{C}}_{\text{o}\_\text{F}}^{\text{in}}$$ (10)

$$\frac{\partial {\text{C}}_{\text{o}\_\text{B}}}{\partial \text{t}}=-\vec{{\text{U}}_{\text{v}}}\cdot \nabla {\text{C}}_{\text{o}\_\text{B}}$$ (11)

$${\text{C}}_{\text{o}\_\text{B}}=4\text{H}\cdot {\text{C}}_{\text{Hb}}\cdot {\text{SO}}_{2}\left({\text{C}}_{\text{o}\_\text{F}}^{\text{in}}\right)$$ (12)

where ${\text{C}}_{\text{o}\_\text{F}}^{\text{in}}$ and C_o_B_ are the free and bound oxygen concentrations inside the vessel, respectively. $\vec{{\text{U}}_{\text{v}}}$ denotes the intravascular blood velocity; H denotes haematocrit obtained from the haemodynamic calculation; C_Hb_ is the haemoglobin concentration within a red blood cell; SO_2_(C_o_F_) is the haemoglobin oxygen saturation [50].

The free oxygen flux across the vessel wall satisfies the Fick's law:

$$\frac{\partial {\text{C}}_{\text{o}\_\text{F}}^{\text{ex}}}{\partial \text{t}}\text{V}=\text{J}\cdot \text{A}$$ (13)

where ${\text{C}}_{\text{o}\_\text{F}}^{\text{ex}}$ is the free oxygen concentration in the tissue space. V and A are the tissue volume and the associated vessel wall area. J is the oxygen flux which is obtained by

$$\text{J}=-{\text{L}}_{\text{p}}\frac{\left({\text{C}}_{\text{o}\_\text{F}}^{\text{ex}}-{\text{C}}_{\text{o}\_\text{F}}^{\text{in}}\right)}{\alpha \text{w}}$$ (14)

where α is the Bunsen solubility coefficient; w is the vessel wall thickness. L_p_ is the vessel wall permeability which is varied in different maturity level of vessel segments.

The interstitial fluid velocity is very slow due to the low interstitial pressure gradient in the tumour region. In fact, Ui is almost 100 times smaller than Uv in value according to the simulation results in our previous model. Therefore, we assume the free oxygen transported through the tissue space is governed only by the oxygen diffusion equation which is not influenced by the interstitial fluid velocity Ui.

$$\frac{\partial {\text{C}}_{\text{o}\_\text{F}}^{\text{ex}}}{\partial \text{t}}=\nabla \cdot \left({\text{D}}_{\text{o}}\nabla {\text{C}}_{\text{o}\_\text{F}}^{\text{ex}}\right)-{\gamma \text{TC}}_{\text{i},\text{j}}$$ (15)

where D_o_ is the tissue oxygen diffusion coefficient and γ is the consumption coefficient.

The initial condition of ECM density is set to be 1 and other chemicals’ concentrations (oxygen, VEGF and MDEs) are 0. No-flux boundary conditions are used in the simulation field. Since chemicals are transported much faster than the characteristic time for cell proliferation and migration, the chemicals’ concentrations are solved to steady state at each time step of the simulation with an inner iteration step of 5s. The oxygen concentration has been normalized to be between 0 and 1 in the result section.

### Tumour cell phenotype

The probabilistic hybrid model for tumour cell growth is based on the previous work [30]. The 3D model is defined on a 100×100×100 grid to cover a 1mm×1mm×1mm volume, so the grid length corresponds approximately to the size of a tumour cell, *i*.*e*. 10μm.

**Fig 3:**
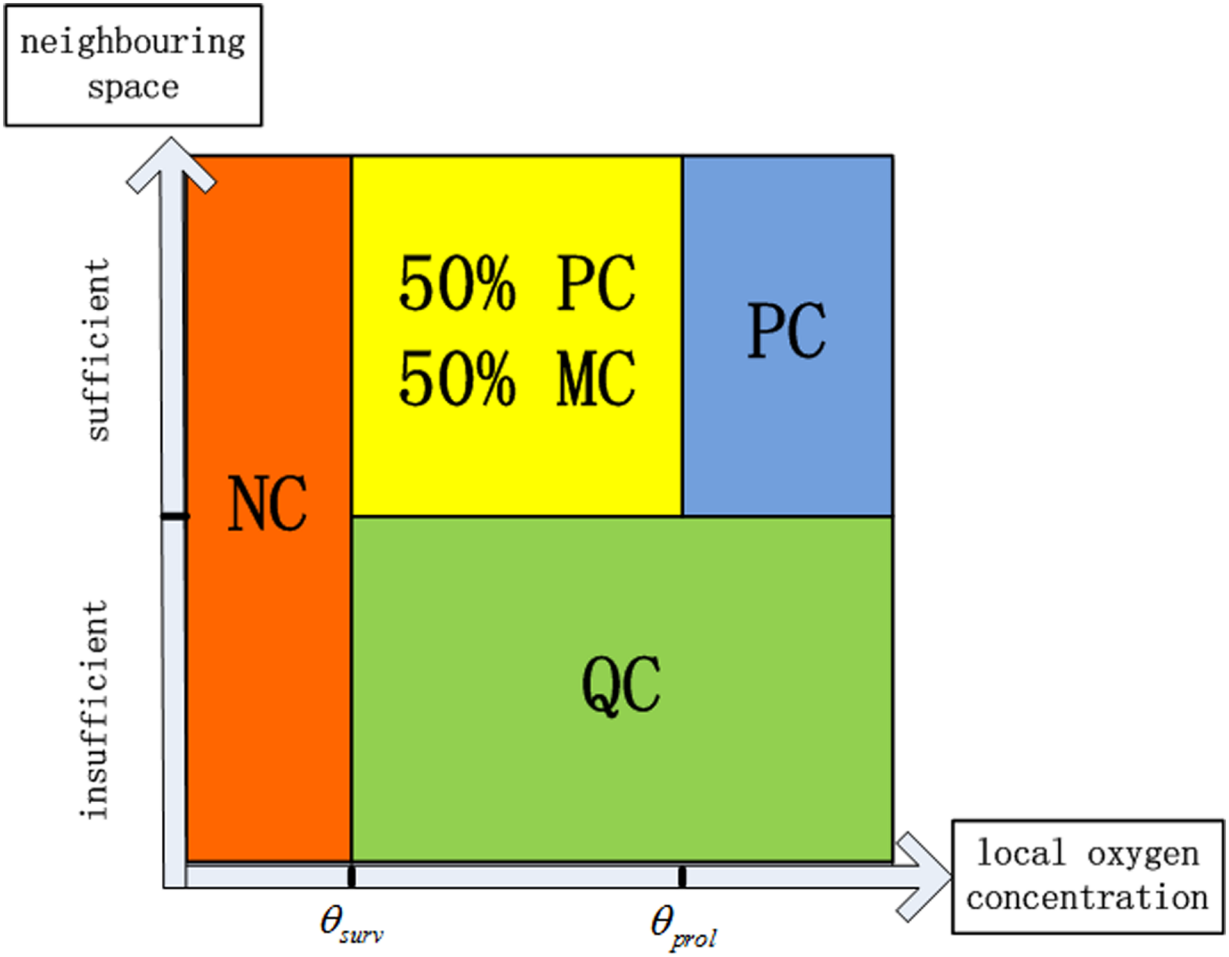
Four phenotypes of glioma cells in the model. The relationships of the four phenotypes of glioma cells with the local microenvironment.

We assumed four different phenotypes of glioma cells: the proliferating cells (PC), the quiescent cells (QC), the necrotic cells (NC) and the migrating cells (MC). Initially, we put 20 proliferating cells in the central area. Two thresholds of oxygen concentration for cell proliferation (θ_prol_) and cell survival (θ_surv_) are introduced to describe the effects of oxygen field on the tumour cell actions. The relationships of the four phenotypes of glioma cells with the local microenvironment are shown in Fig 3.

To be specific, if the local oxygen ($C_{o}^{ex}\geq \theta_{\text{prol}}$) and a neighbouring space is available, a tumour cell will proliferate into two daughter cells with a probability, defined as T_age_/T_TC_. T_TC_ is the tumour cell proliferation time (set to be 9 hours, equals to 6 time steps). T_age_ is the tumour cell age, ranging from 1 to T_TC_ and with an incremental 1 in each simulation time step. One of the two daughter cells will replace the parent cell and the other cell will move to a neighbouring node space. When the local oxygen concentration at a tumour site is less than the cell survival threshold θ_surv_, the tumour cell is marked as a necrotic cell and will not be revisited at the next time step. A necrotic cell has a probability of 20% to disappear and release the space for a glioma cell or an endothelial cell if it stays necrotic for more than 45 hours (30 time steps). When a tumour cell satisfies the survival condition but there is no neighbouring space for it to proliferate, it will go quiescent. In the current model, there is no time limit for a cell to stay in the quiescent state. When the neighbouring space of one quiescent cell has been released, the quiescent cell will turn back into a proliferating cell if the local oxygen supply is sufficient.

To cope with the glioma cell migration ability, a specific phenotype, called the migrating cell (MC), was defined. When local oxygen level is higher than θ_surv_ but lower than θ_prol_ and a space is available, a proliferating cell has a probability (50%) to become a migrating cell, and will migrate to a neighbouring space which has the highest oxygen concentration. It was also assumed that the migrating cells adjacent to the pre-existing vessel wall have higher probability of moving in the longitudinal direction (vessel axial direction) than the radial direction. The migration speeds of the two directions are the same, *i*.*e*., 10μm per time step. After a migrating cell completes its movement, the space it originally occupied will be released. Each phenotype of tumour cell has a different coefficient of oxygen consumption rate and the production rate of VEGF and MDEs [30] (Table 2).

**Table 2:** Parameters of different phenotypes of glioma cells.

Phenotypes	MDE production	VEGF production	Oxygen consumption
Migrating cells (M)	2*μ*_T_	*χ* × 4	2*γ*
Proliferating cells (P)	*μ*_T_	*χ*	*γ*
Quiescent cells (Q)	*μ*_T_/5	*χ* × 2	*γ*/2
Necrotic cells (N)	*μ*_T_/10	*χ* × 4	*γ*/4

### Vessel co-option, remodelling, collapse and angiogenesis

**Fig 4:**
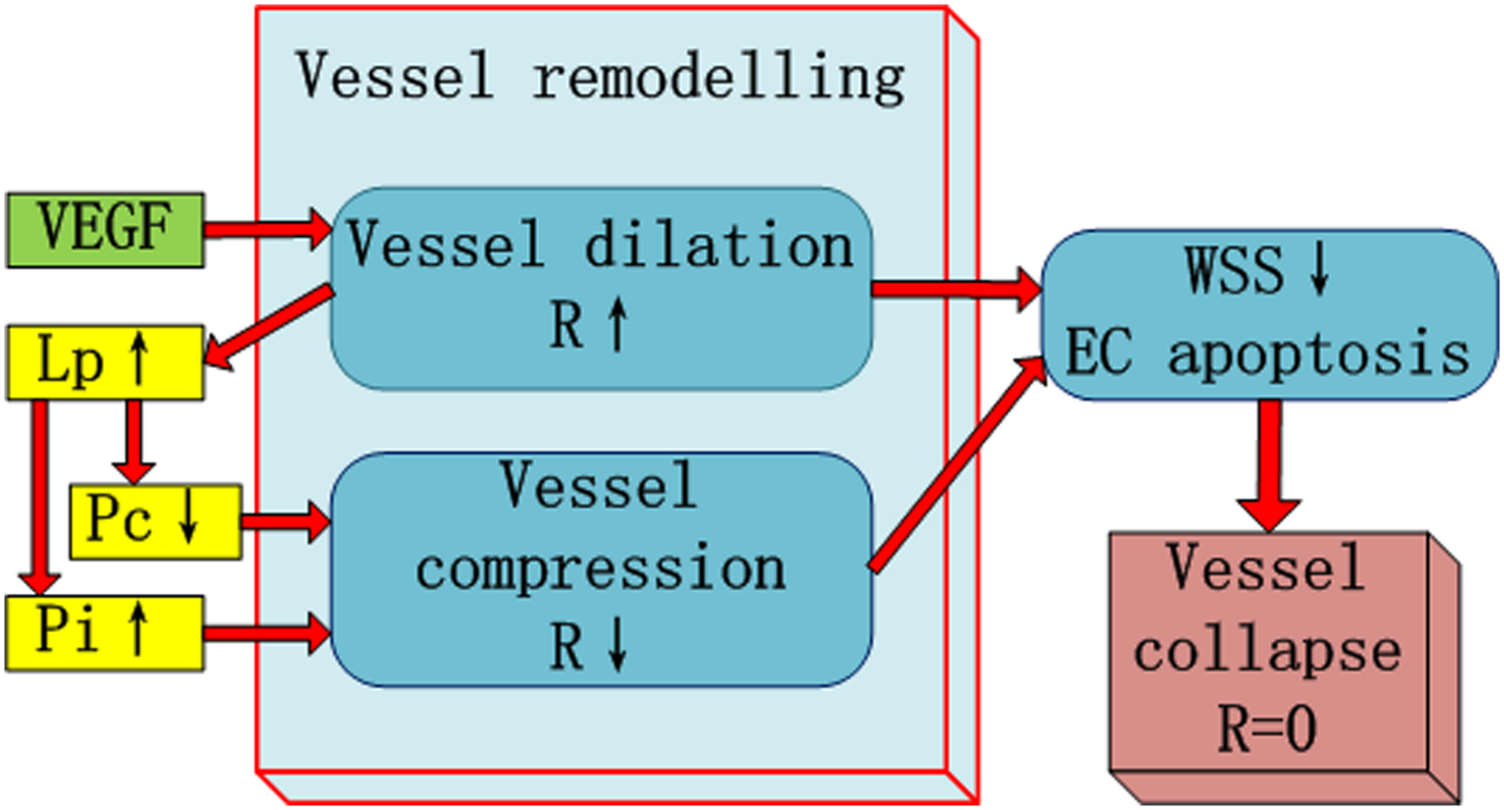
Modelling of vessel remodelling and collapse. The schematic diagram for the interactions between vessel remodelling and collapse with the microenvironment.

Experimental and clinical studies both revealed that microvessel diameter increases in response to growth factors. Döme *et al* [10] found that even a single tumour cell can induce radical changes in the host tissue vasculature in a mouse model of glomeruloid angiogenesis. In our model, we consider vessel dilation as the first sign of a pre-existing vessel becoming an immature vessel. However, vessel dilation and compression are a coupled phenomena in our model as shown in Fig 4. In brief, a high local VEGF concentration will cause a vessel segment inside the tumour to be dilated which will cause an increase in vessel wall permeability and a decrease in vessel collapsing pressure. In this way, vessel may be compressed due to the increased interstitial pressure. A vessel with a changed diameter will influence local wall shear stress (WSS). We assume that a vessel segment will collapse if the local WSS is too low for a long period.

A vessel segment inside the tumour that has a VEGF concentration larger than a threshold θ_VEGF_ will increase its radius R with the rate of 0.40μm/h which will stop when the vessel radius reaches the maximum value of R_max_ = 10 μm. At the same time, the permeability of the vessel wall L_p_ is increasing in a dilation vessel, and satisfies

$${\text{L}}_{\text{p}}=\{\begin{array}{c} {\text{L}}_{\text{P}}^{\text{T}}\left(\frac{\text{R}}{{\text{R}}_{\text{max}}}\right),\;\text{immature vessel}\\ {\text{L}}_{\text{P}}^{\text{N}},\;\text{mature}\;\text{vessel} \end{array}$$ (16)

where ${\text{L}}_{\text{p}}^{\text{N}}$ is the initial value of L_p_ referred to the vessel permeability value in the normal tissue; ${\text{L}}_{\text{P}}^{\text{T}}$ is the maximum value of L_p_ according to the experiments of vessel permeability value in a tumour microvessel.

The pressure value that will cause a vessel to collapse is defined as P_c_ which represents the ability of a vessel segment remaining structurally intact under the trans-wall pressure difference. In this study, initial P_c_ values were predefined for each vessel according to the Strahler order of the vessel segment in the pre-existing vessel network. The vessel with the larger diameter has a higherinitial P_c_. When the local microenvironment was changed by the embedded glioma cells, Ang-2 is up-regulated in co-opted vessels, causing the destabilization of the vessel wall, *i*.*e*., the detachment of pericytes from the endothelial tube [6]. Since the Ang-2 concentration and pericyte density are not included in this model, we assume that P_c_ decreases with increasing permeability of vessel wall Lp in the immature vessel.

$${\text{P}}_{\text{c}}={\text{P}}_{\text{c}}^{\text{min}}\left(\frac{{\text{L}}_{\text{p}}^{\text{T}}}{{\text{L}}_{\text{p}}}\right)$$ (17)

where ${\text{P}}_{\text{c}}^{\text{min}}$ is the smallest collapse pressure that an immature vessel segment can have, and set to be 0.5P_c_ of different Strahler orders.

For a pre-existing vessel, once vessel dilation occurs, the vessel segment is treated as an immature vessel with increased L_p_ and decreased P_c_. In the simulation, vessel wall compliance is defined by the radius changing under the influence of intravascular and interstitial pressures and collapse pressure based on the empirical equation of Netti *et al* [51].

$$\text{R}=\{\begin{array}{c} {\text{R}}_{0}{\left(\frac{{\text{P}}_{\text{v}}-{\text{P}}_{\text{i}}+{\text{P}}_{\text{c}}}{\text{E}}\right)}^{\text{b}},\;\text{immature}\;\text{vessel}\\ {\text{R}}_{0},\;\text{mature}\;\text{vessel} \end{array}$$ (18)

where R_0_ is the origin radius of the capillary; b is the compliance exponent; E is the compliance coefficient.

The three variables R, L_p_ and P_c_ are fully coupled in the model. In the simulation of vessel remodelling, we first solved Eq 16 for all immature vessels, *i*.*e*., dilated vessels, to obtain the values of L_p_. Then we perform the haemodynamic calculation with updated L_p_ to obtain P_i_, and also solved Eq 17 to obtain P_c_. Finally we updated vessel radius R for the iteration by P_i_ and P_c_ according to Eq 18.

Based on the above equations, when the vessel segment becomes immature, L_p_ will increase which causes lower P_c_, and consequently P_i_ will increase, both of the changes can cause vessel compressing. A compressed vessel, on the other hand will induce a higher flow resistance, lower flow which will then decrease the wall shear stress (WSS) level for the vessel. Vessel collapse will occur by either WSS criteria(as described below) or a significant reduced R.

As defined in our previous work and others’ that vessel will collapse due to a long period of low WSS status in which the apoptosis of EC dominate the collapse process [28–30]. WSS is used to estimate this kind of vessel regression. The WSS of a vascular segment can be calculated as

$$\tau =\frac{\Delta P_{V}\cdot R}{2\Delta l}$$ (19)

We assume that a circulated vessel, which is surrounded by the TCs, will collapse with a pre-defined probability if the WSS value in the vessel is <1/2f_0_ where f_0_ is the mean WSS value in the vessels of Strahler order 3 in the entire model. The probability is assumed to be proportional to the duration of low WSS in the vessel, *i*.*e*., the longer the vessel experiences the low WSS, the more likely the vessel is to collapse if the criterion is satisfied.

A 3D hybrid discrete-continuum angiogenesis model was adopted to investigate the EC migration and proliferation through random motility, chemotaxis in response to VEGF distributions and haptotaxis in response to the local ECM density [13]. Each EC occupies one grid as the tumour cell. Endothelial sprouting is only allowed in immature vessels, and the endothelial cell distribution was updated based on the equation

$$\frac{\partial \text{e}}{\partial \text{t}}={\text{D}}_{\text{e}}\nabla^{2}\text{e}-\nabla \cdot \left(\frac{\phi_{\text{c}}}{1+{\sigma \text{C}}_{\text{v}}}\text{e}\nabla {\text{C}}_{\text{v}}+\phi_{\text{h}}\text{e}\nabla {\text{C}}_{\text{f}}\right)$$ (20)

where e is the EC density, D_e_, ϕ_c_, ϕ_h_ are EC diffusion, chemotaxis and haptotaxis coefficients, respectively. ECs are allowed to move along the six directions in a 3D dimension space. All neo-vasculatures are assumed to be immature vessels and Strahler order 1.

### Simulation framework: working procedure

**Step 0.** Initialize.

** Step 0.1** Create the 3D pre-existing vessel network, and initialize the model parameters.

** Step 0.2** Put 20 proliferating cells with random ages from T = 1 to T_TC_, near the capillaries of Strahler order 1.

** Step 0.3** Set up fluid flow boundary conditions.

**Step 1.** Calculate haemodynamics (Eqs 1–6), and obtain the flow information.

**Step 2.** Calculate the chemical’s concentration field.

** Step 2.1** Solve the Eqs 7–9 to obtain the concentration distribution of ECM, MDE and VEGF.

** Step 2.2** Solve the Eqs 10–12 to obtain the oxygen transport inside the vessel using the intravascular blood velocity $\vec{{\text{U}}_{\text{v}}}$ and haematocrit H from Step 1.

** Step 2.3** Solve the Eqs 13 and 14 to obtain the free oxygen flux across the vessel wall.

** Step 2.4** Solve the Eq 15 to obtain the free oxygen transported through the tissue space.

** Step 2.5** Update the oxygen concentration field through the simulation domain.

**Step 3.** Determine the behaviour of tumour cells according to the local oxygen concentration, the available space and the cell age, and update the tumour cell distribution.

**Step 4.** Update the vessel network.

** Step 4.1** Vessel co-option, and vessel radius and permeability changes (Eq 16) in immature vessels.

** Step 4.2** Endothelial cells migrate and proliferate to form neo-vasculature (Eq 20).

** Step 4.3** Vessel remodelling according to Eqs 17 and 18 and update the Pc and radius of vessel segments.

** Step 4.4** Certain vessel collapse based on the R changing and WSS criterion (Eq 19).

**Step 5.** Go to Step 1.

Each time step increment (T = T+1) corresponds to 1.5 hour. We will use non-dimensional time unit instead of hours in the following results. The parameter values of the baseline model are listed in Table 3.

**Table 3:** Parameter values used in the simulation.

Parameter	Value	Description	Reference
Δl	10μm	Lattice constant	
σ_T_	0.82	Average osmotic reflection coefficient for plasma proteins	Baxter & Jain (1989) [52]
π_v_	20mmHg	Colloid osmotic pressure of plasma	Baxter & Jain (1989) [52]
π_i_	15mmHg	Colloid osmotic pressure of interstitial fluid	Baxter & Jain (1989) [52]
K	4.13×10^−8^cm^2^/mmHg s	Hydraulic conductivity coefficient of the interstitium	Baxter & Jain (1989) [52]
S/V	200cm^-1^	Surface area per unit volume for transport in the interstitium	Baxter & Jain (1989) [52]
*D*_*m*_	10^−9^*cm*^2^*s*^−1^	MDE diffusion coefficient	Anderson. (2005) [53]
*δ*	1.3×10^2^*cm*^3^*M*^−1^*s*^−1^	ECM degradation coefficient	Cai et al. (2011) [30]
*μ*_T_	1.7×10^−18^*Mcells*^−1^*s*^−1^	MDE production by TC	Cai et al. (2011) [30]
*μ*_E_	0.3×10^−18^*Mcells*^−1^*s*^−1^	MDE production by EC	Cai et al. (2011) [30]
*λ*	1.7×10^−8^*s*^−1^	MDE decay coefficient	Anderson. (2005) [53]
*D*_*v*_	2.9×10^−7^*cm*^2^*s*^−1^	VEGF diffusion coefficient	Anderson & Chaplain (1998) [13]
*χ*	10^−17^*Mcells*^−1^*s*^−1^	VEGF production by TC	Alarcón et al. (2006) [26]
*ξ*	10^−3^*cm*^−3^*s*^−1^	VEGF production in ECM	Cai et al. (2011) [30]
*ε*	10^−20^*Mcells*^−1^*s*^−1^	VEGF consumption by EC	Alarcón et al. (2006) [46]
*θ*	10^−8^*s*^−1^	VEGF decay coefficient	Alarcón et al. (2006) [46]
α	1.27×10^−15^μmol/(μm^3^mmHg)	Bunsen solubility coefficient	Fang et al. (2008) [50]
*D*_*o*_	10^−5^*cm*^2^*s*^−1^	Oxygen diffusion coefficient	Anderson. (2005) [53]
*γ*	6.25×10^−17^*Mcells*^−1^*s*^−1^	Oxygen consumption coefficient	Anderson. (2005) [53]
${\text{L}}_{\text{p}}^{\text{T}}$	2.8×10^−7^cm/mmHg s	Vessel permeability in tumour tissue	Baxter & Jain (1989) [52]
*D*_*e*_	10^−9^*cm*^2^*s*^−1^	EC diffusion coefficient	Anderson & Chaplain (1998) [13]
*ϕ*_*c*_	2.6×10^3^*cm*^2^*M*^−1^*s*^−1^	EC chemotaxis coefficient	Anderson & Chaplain (1998) [13]
*ϕ*_*h*_	10^3^*cm*^2^*M*^−1^*s*^−1^	EC haptotaxis coefficient	Anderson & Chaplain (1998) [13]
E	6.5mmHg	Vessel compliance coefficient	Netti et al. (1996) [51]
b	0.1	Vessel compliance index	Netti et al. (1996) [51]

## Results

### General features of GBM growth

**Fig 5:**
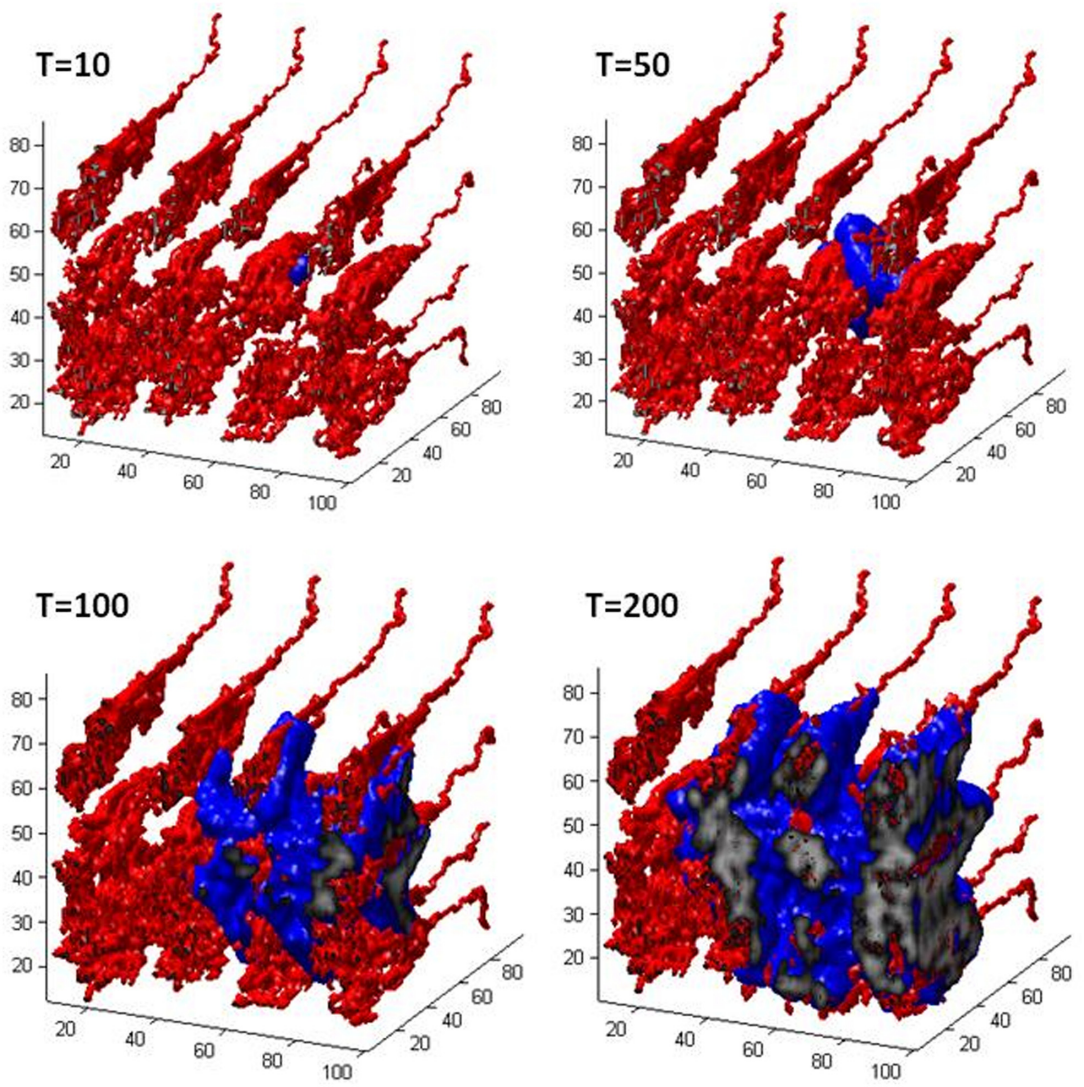
3D global pictures of GBM distribution and vessel network at different time phases during the growth period. The red tubes represent microvessels. The blue region shows the invasion area of GBM to the surrouding tissue, while the grey regions represent the necrotic cores of the tumour.

Fig 5 shows 3D global pictures of the GBM distribution and vessel network at different time phases during the growth period. The red tubes are the capillaries. The blue region shows the invasion area of GBM to the surrouding tissue, while the grey regions represent the necrotic cores of the tumour. It clearly indicates that the tumour develops around well perfused regions at the early phases of growth. Althrough not presented, there is hardly any tumour cell growth in the region with no pre-existing vessel even at T = 150.

**Fig 6:**
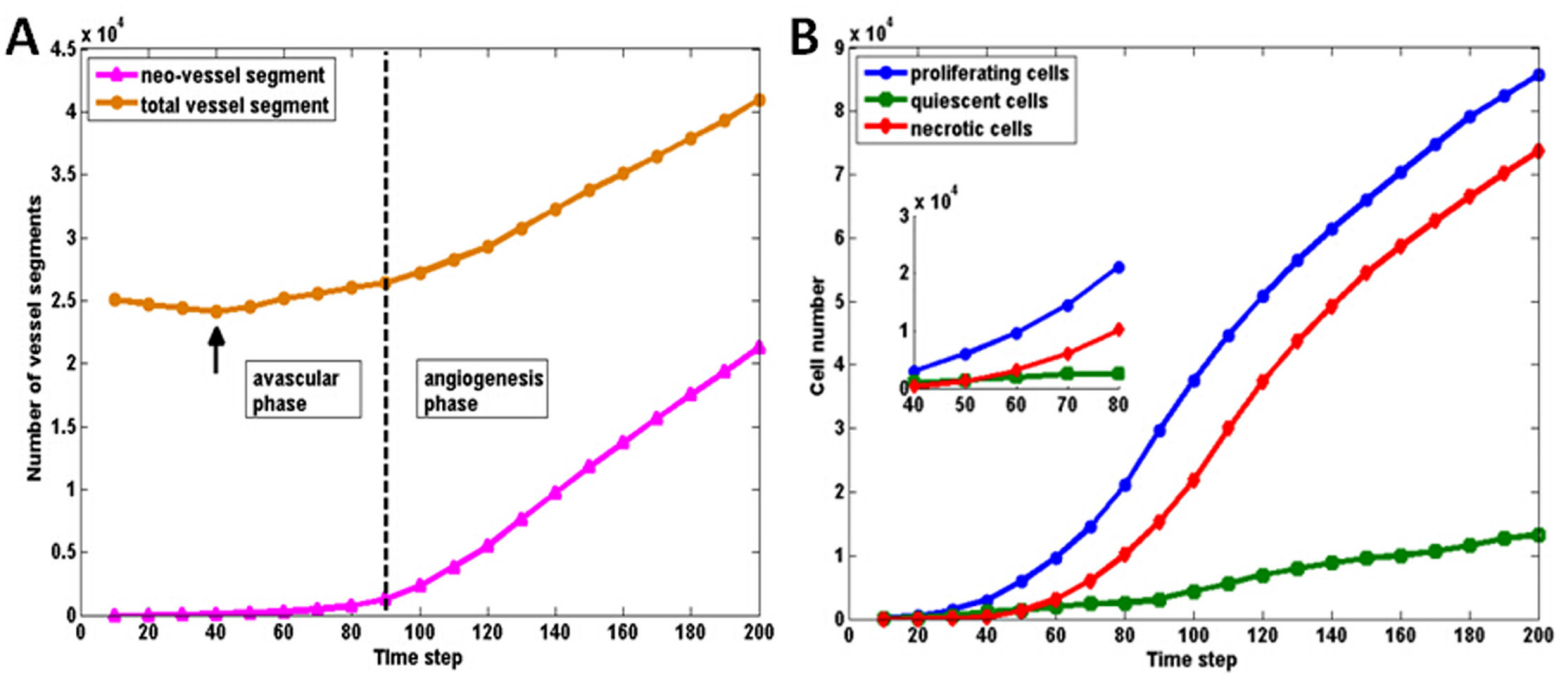
Growth curves of the baseline model. A. The growth curves of angiogenic vessels and total tumour vessels in the baseline model. The arrow shows the decrease of vessel segments due to the co-option and remodelling of pre-existing vessels at the early stage. The broken line shows the starting point of angiogenesis which is defined when the neo-vessels account for 5% of total vessel segments. B. The growth curves of different phenotypes of tumour cells in the baseline model. A close up view at the early phase from T = 40 to T = 80 is shown in the inserted panel.

The growth history curves in Fig 6A show the development history and characteristics of tumour cells and vessel segment more directly. At the early growth stage (T<60), there are limited neo-vasculature vessels which suggests that the pre-existing vessel network supplied sufficient nutrients to satisfy the requirement of an early GBM cluster. However, the total number of vessel segments decreased at around T = 40 due to the collapse and remodelling of pre-exsting vessels (arrows in Fig 6A). To have a better comparison, the starting point of angiogenesis for a simulation is defined when the neo-vessels account for 5% of total vessel segments (the broken line shown in Fig 6A). After T = 100, the angiogenesis phase occurs due to the increasing hypoxic area. The tumour is then in an accelerating development phase. When analysing the TC growth curve in detail (Fig 6B), it can be seen that the number of quiescent cells is normally small, about 10% of total number of TCs, while the growth curves for proliferating cells and necrotic cells are parallel with a difference of about 10k. Also in the early phase (as shown in the close-up view from T = 40 to T = 80 in Fig 6B), the number of quiescent cells is slightly higher than the number of necrotic cells at T = 40, which is due to the sufficient oxygen supply but limited available space in the microenvironment. However, the avascular tumour rapidly uptakes oxygen from surrounding tissues. As oxygen levels drop, the number of the necrotic cells overtakes the quiescent cells at about T = 60.

### Pseudopalisade formation

**Fig 7:**
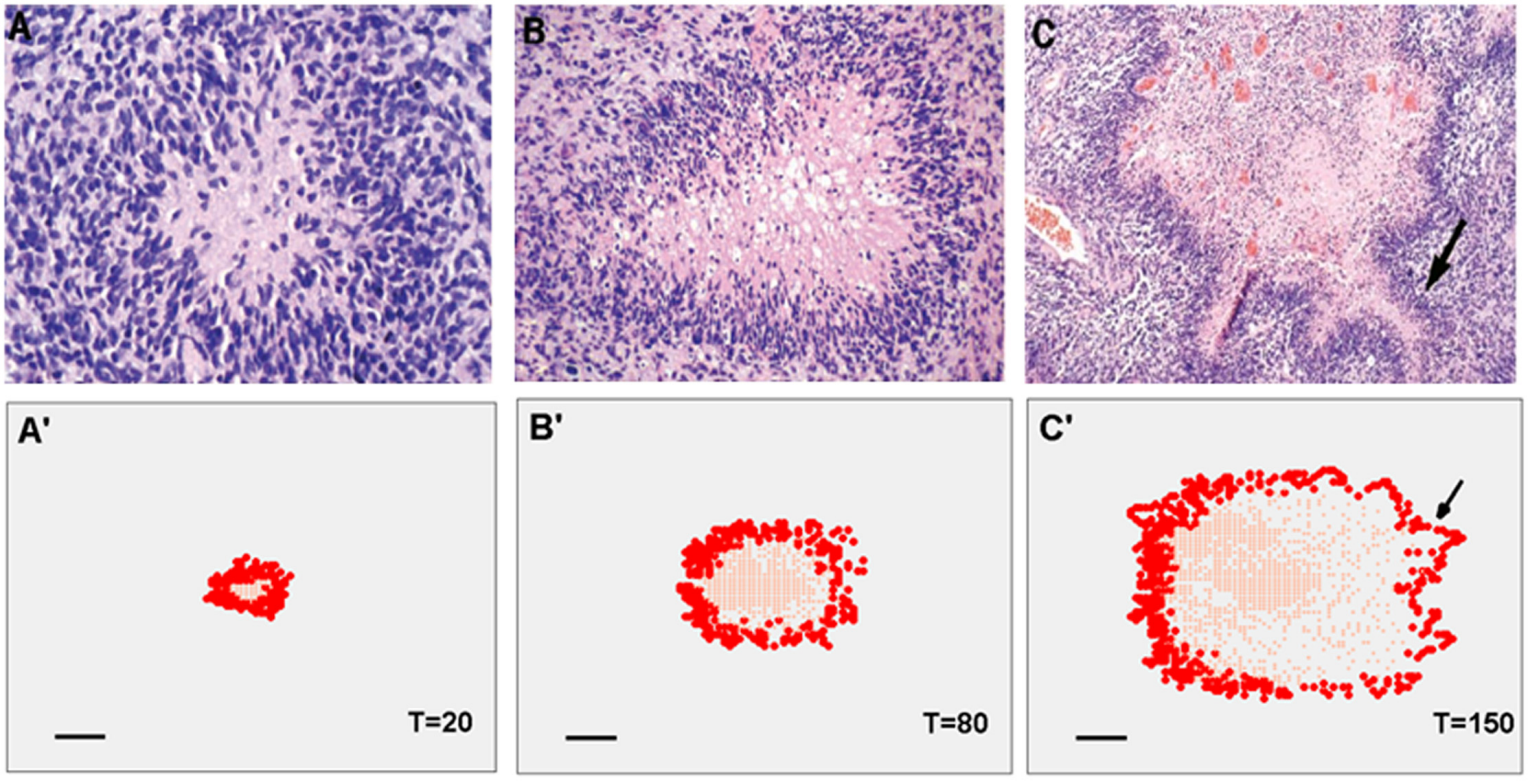
Comparison of experimental and simulation results of pseudopalisade formation in GBM. The Histopathologic pictures of pseudopalisades in GBM (A, B and C) [4]. A, narrow pseudopalisades (<100 micron wide). B, medium-sized pseudopalisades (200–400 micron). C, large pseudopalisade (>500 micron). The simulation results of pseudopalisades with different sizes (A', B' and C'). Red dots represent the proliferating and migrating tumour cells gathered at the tumour periphery. Light red dots represent the quiescent cells. Arrows in C and C' show the outpouchings of pseudopalisades. Scale bar: 100 micron.

**Fig 8:**
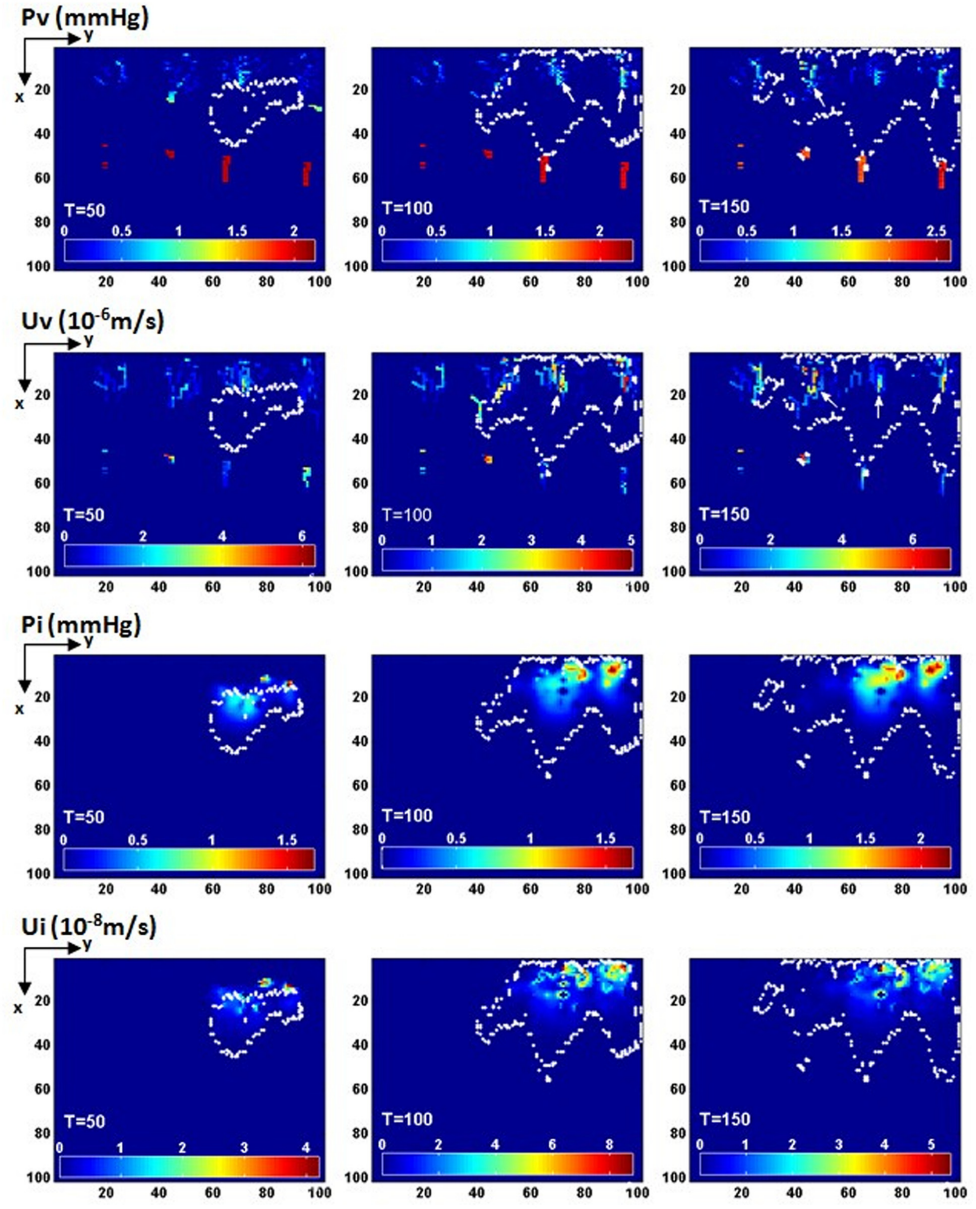
Simulation results of haemodynamics. The haemodynamic information including the intravascular pressure Pv, the intravascular blood velocity Uv, the interstitial pressure Pi and the interstitial fluid velocity Ui at three different time points (T = 50,100,150, plane z = 50). The white dots show the boundary of the GBM. The white arrows point to the vessels which provide main blood perfusion in the GBM.

Fig 7 shows the comparison of experimental and simulation results of pseudopalisade formation in GBM. At the early stage (T = 20), the proliferating cells (PCs) and migrating cells (MCs) (shown as red dots in Fig 7A') gathered at the tumour periphery and formed hypercellular zones, while quiescent cells (shown as light red dots in Fig 7A') mostly distributed in the tumour centre. This is consistent with the histopathologic observation [4] that narrow pseudopalisades usually have hypercellular zones but lack central necrosis (Fig 7A). Due to the high oxygen consumption of PCs and MCs, these large numbers of PCs and MCs induce serious hypoxia in the surrounding region. With the tumour growth, the pseudopalisades undergo central necrosis (blank holes in the tumour centre in Fig 7B and 7B'). It is worth mentioning that there are still a few functioning vessels inside the pseudopalisades (see Fig 8). With the co-option and remodelling of pre-existing vessels induced by the invasion of GBM cells, some vessels become immature and surrounded by tumour cells to be intra-tumour vessels. As a consequence, the migrating cells invaded health tissue further, increasing the area of pseudopalisades and forming some outpouchings of pseudopalisades (arrows in Fig 7C and 7C'). These outpouchings could give rise to smaller pseudopalisades resembling the narrow pseudopalisades as shown in Fig 7A and 7A'.

### Haemodynamics and pre-existing vasculature remodelling

The haemodynamic information including the intravascular pressure Pv, the intravascular blood velocity Uv, the interstitial pressure Pi and the interstitial fluid velocity Ui at three different time points (T = 50,100,150, plane z = 50) are shown in Fig 8. Pv in the vessels of Strahler order 3 maintains a relatively high level compared to the capillaries. The high Pv and Uv region inside the tumour both show heterogenous distributions. It confirms the heterogeneous blood perfusion in malignant tumours observed from the experiments [54]. At the same time, some vessel segments inside the tumour in the capillary network show high flow rates(white arrows)which make these vessels the main providers of local oxygen in the central region of the GBM. The interstitial pressure Pi in the interior of the tumour generally increases with tumour growth, and the highest Pi occurred at the tumour central area, which is one of the typical pathological features of GBM. The peritumoural oedema is caused by the intense and dysfunctional vascularization in GBM, and will lead to the heterogeneous delivery of oxygen and drugs. Ui is very slow inside the tumour (100 times smaller than Uv in value).

**Fig 9:**
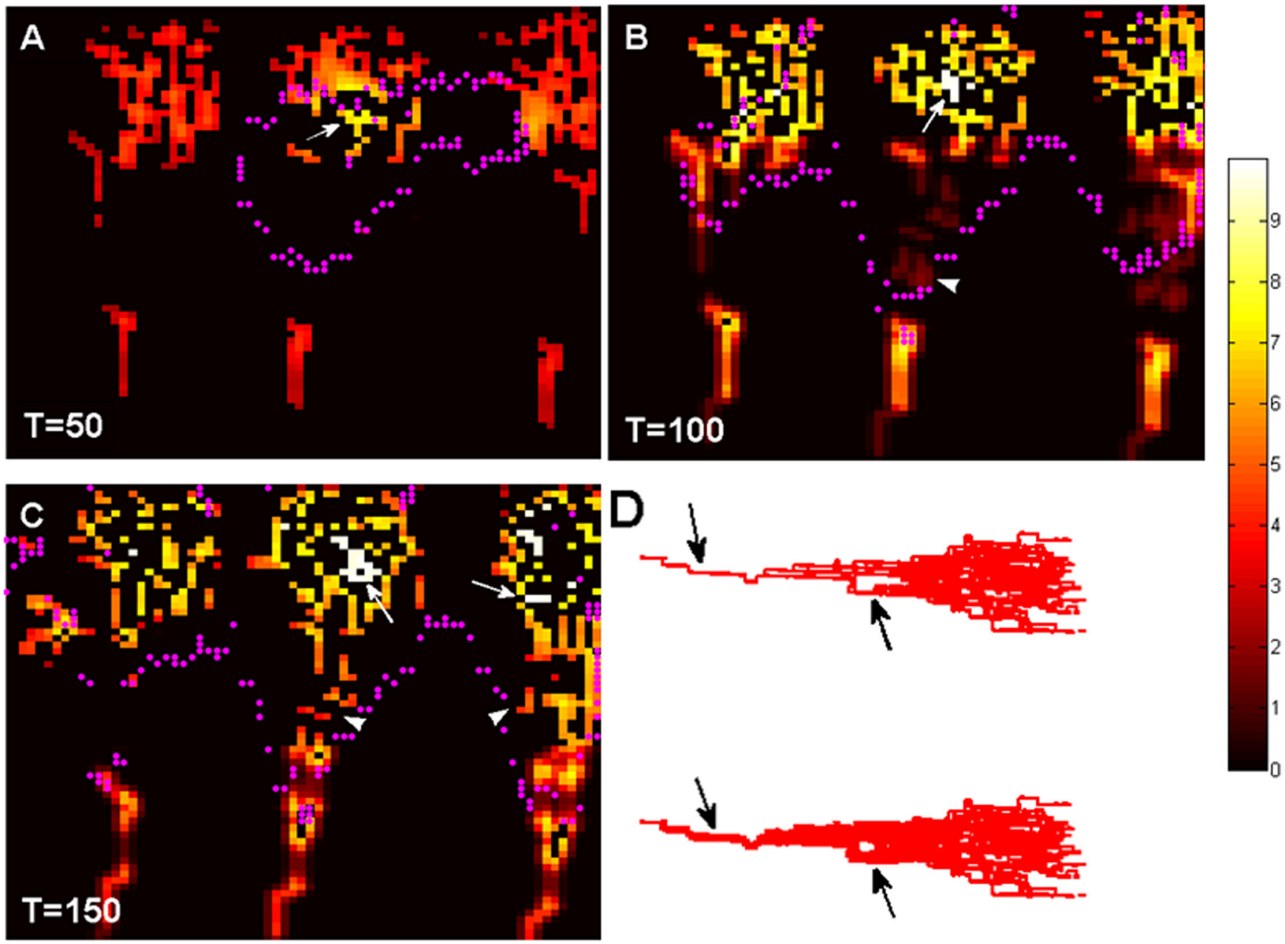
A zoom-in view of the vessel remodelling in the tumour region. Microvessel radius (R) is represented by different colour (unit:μm). Pink dots represent the approximate boundary of GBM invasion. White arrows indicate the vessel dilation (A, B, C). White arrowheads indicate the neo-vasculatures. D. 3D view of a cluster of capillaries before (top) and undergo (bottom) remodelling. Note the dilated vessels indicated by arrows.

The pre-existing vessel co-option, remodelling and collapse can be seen in Fig 9. Microvessel radius (R) is coded by colour (unit:μm). Pink dots represent the approximate boundary of GBM invasion. At the early stage (T = 50), vessels far away from the tumour show uniform distribution in vessel diameter. However, the vessels inside the tumour have already undergo dilation and remodelling (white arrow in Fig 9A). At T = 100, microvessel density in the tumour centre reduced significantly, at the same time, few dilated vessels (white arrow in Fig 9B) left in the tumour centre become the main perfusion path for transport of oxygen and nutrients to the growing tumour. After further remodelling process, the dilated functional vessels increase in number in this stage (also shown in 3D view in Fig 9D). There were scattered angiogenic capillaries (arrowhead in Fig 9B) at T = 100; some of these vessels will undergo maturation and compose blood perfusion pathway to the tumour centre (arrowheads in Fig 9C). It is worth mentioning that the large variation in vessel diameters will increase the flow resistance in tumours microvasculature. As a result, tumour blood flow is unevenly distributed (as shown in Fig 8), which causes abnormal microenvironment in tumours.

### Influence of initial glioma cell location on the GBM growth

**Fig 10:**
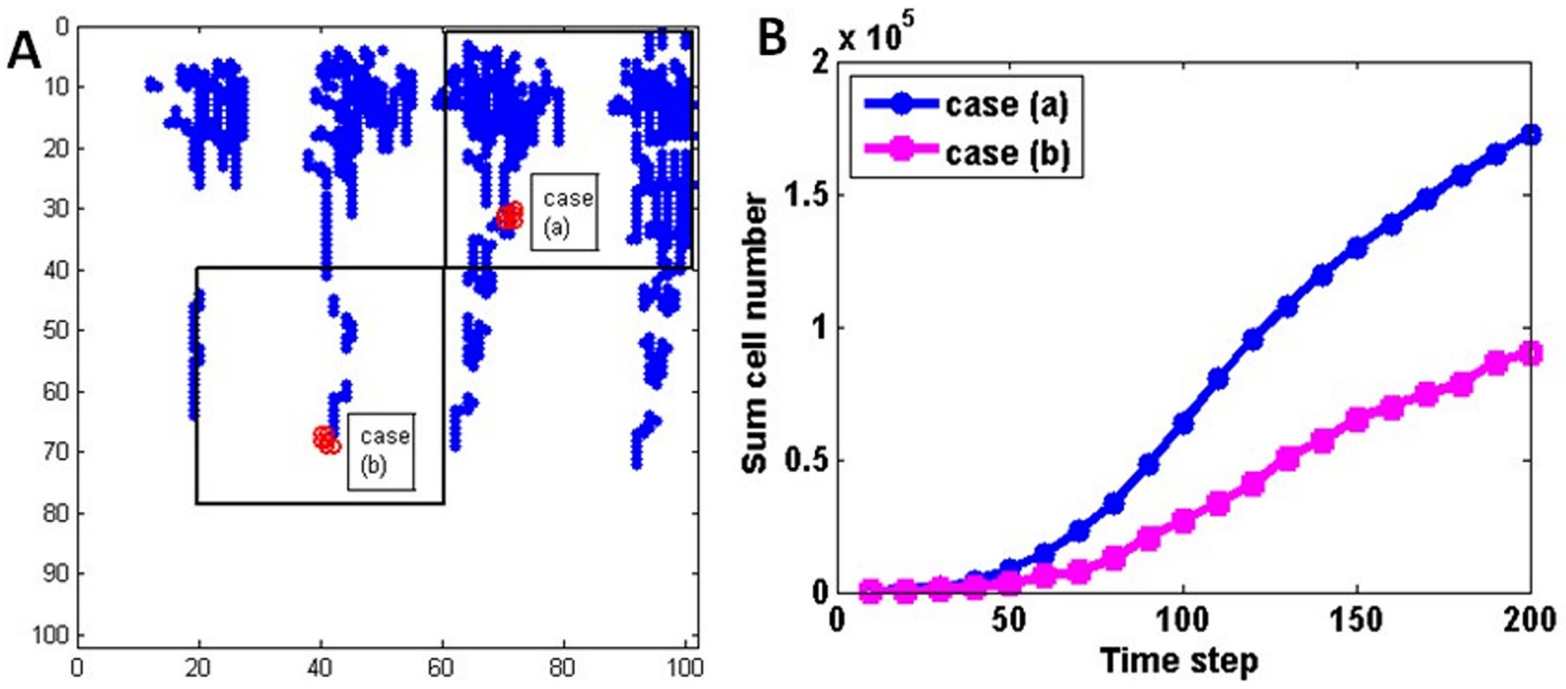
Influence of initial glioma cell location on the simulation. A. 20 proliferating GBM cells were planted at a relatively blood-supply-sufficient microenvironment (case (a)) and a blood-supply-deficient region (case (b)). B. The GBM cell growth history curves of above two cases.

**Fig 11:**
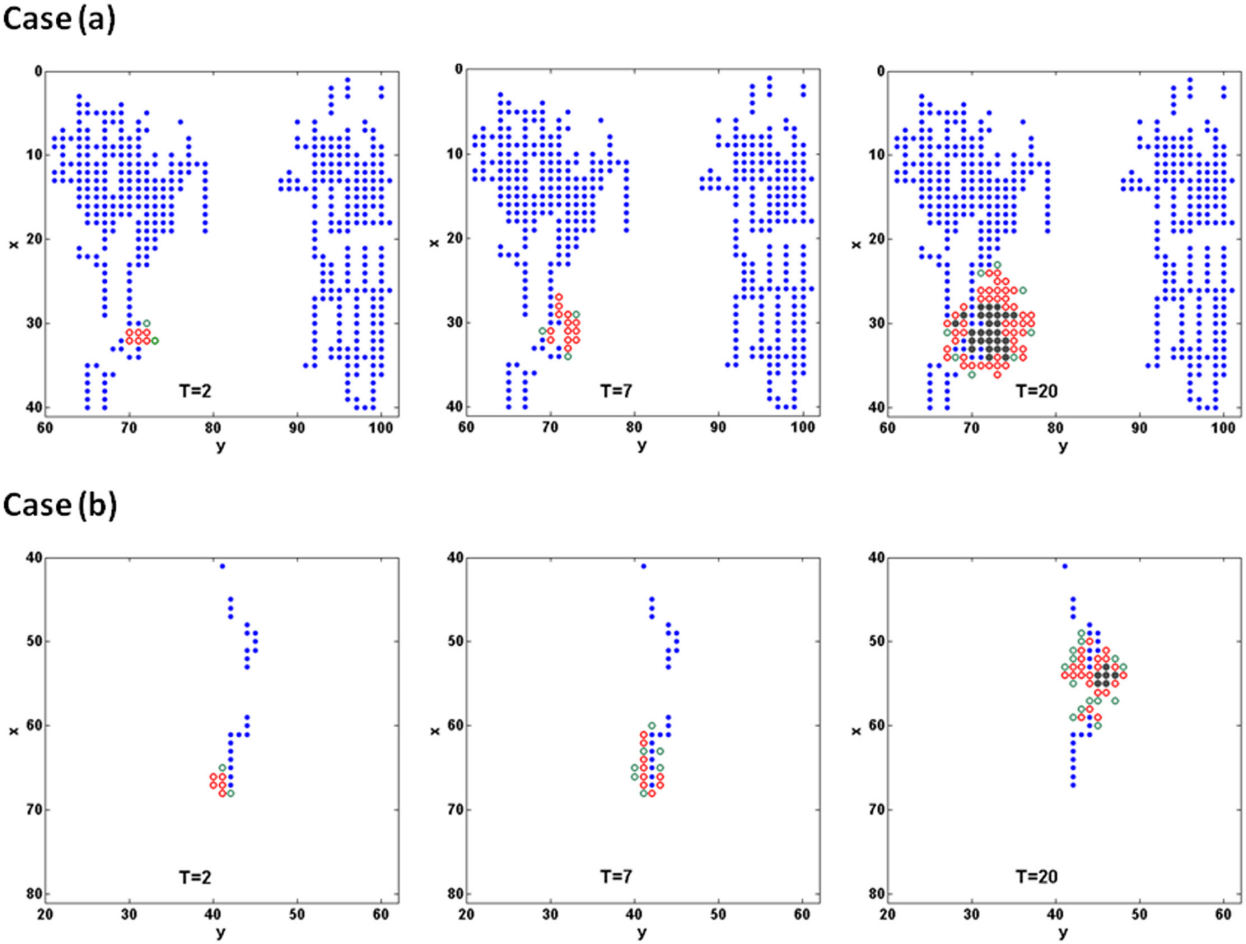
The enlarged view of microvessel and TC distribution at different early growing time of the two cases in Fig 10A. Blue dots represent the vessels, red, green circles and grey dots represent proliferating, migrating and quiescent tumour cells, respectively.

One of the major improvements of the present model compared to our previous ones is the inclusion of tumour cell migration to investigate the malignant characteristics of GBM. To show the impact of cell migration in different metabolic environments to tumour growth, a comparison study was performed by planting GBM cells at regions of different pre-existing blood perfusion conditions (Fig 10). In the case presented in previous sections, 20 proliferating GBM cells were planted at a relatively blood-supply-sufficient microenvironment (Fig 10A, case (a)). Now in an additional simulation case (Fig 10A, case (b)), 20 proliferating GBM cells were planted in a blood-supply-deficient region. The GBM cell growth history curves are shown in Fig 10B, and an enlarged view of microvessel and TC distribution at different times during early GBM growth are shown in Fig 11. In Fig 11, blue dots represent the vessels, red, green circles and grey dots represent proliferating, migrating and quiescent tumour cells, respectively. It was found, for the case (a), that the GBM cells proliferate near the initial site and form an avascular multicellular aggregating region. Since viable cells are more likely to proliferate in the oxygen-rich environment ($C_{o}^{ex}\geq \theta_{\text{prol}}$), few migrating cells are found at the tumour periphery, which suggests that cell migration plays a minor role in this case. However, in a blood-supply-deficient microenvironment (*i*.*e*., case (b)) where the local oxygen supply cannot satisfy the GBM growth, the cells manage to move along the host vessels for a long distance to gain more blood supply, and eventually proliferate at the new site to form the GBM aggregation. The distance from the primary site to their destination can be as long as 100μm which is ten times the cell diameter. This result is consistent with the pathological experiments [9] on the initiation of primary micro-tumour and micro-metastases.

The growth curves of cases(a) and (b) are shown in Fig 10B. In case (b), the total number of glioma cells is significantly smaller compared to case (a), especially in the late stage of tumour growth (T>100). This suggests that cell migration may influence not only the location of the GBM metastasis site, but also the aggressive size of GBM.

### Influences of newly added factors on the model

We carried out additional simulations to investigate the influences of assumptions and newly added simulation factors on the current model, including (a) the new phenotype of migrating cells, (b) the dynamic feedbacks of vessel remodelling and (c) the flow-dependent oxygen calculation. In the following results, three test simulations were performed with the same conditions of the basic model, except the cut-off of the above three feedbacks in the basic coupled model respectively.

#### Cell migration

**Fig 12:**
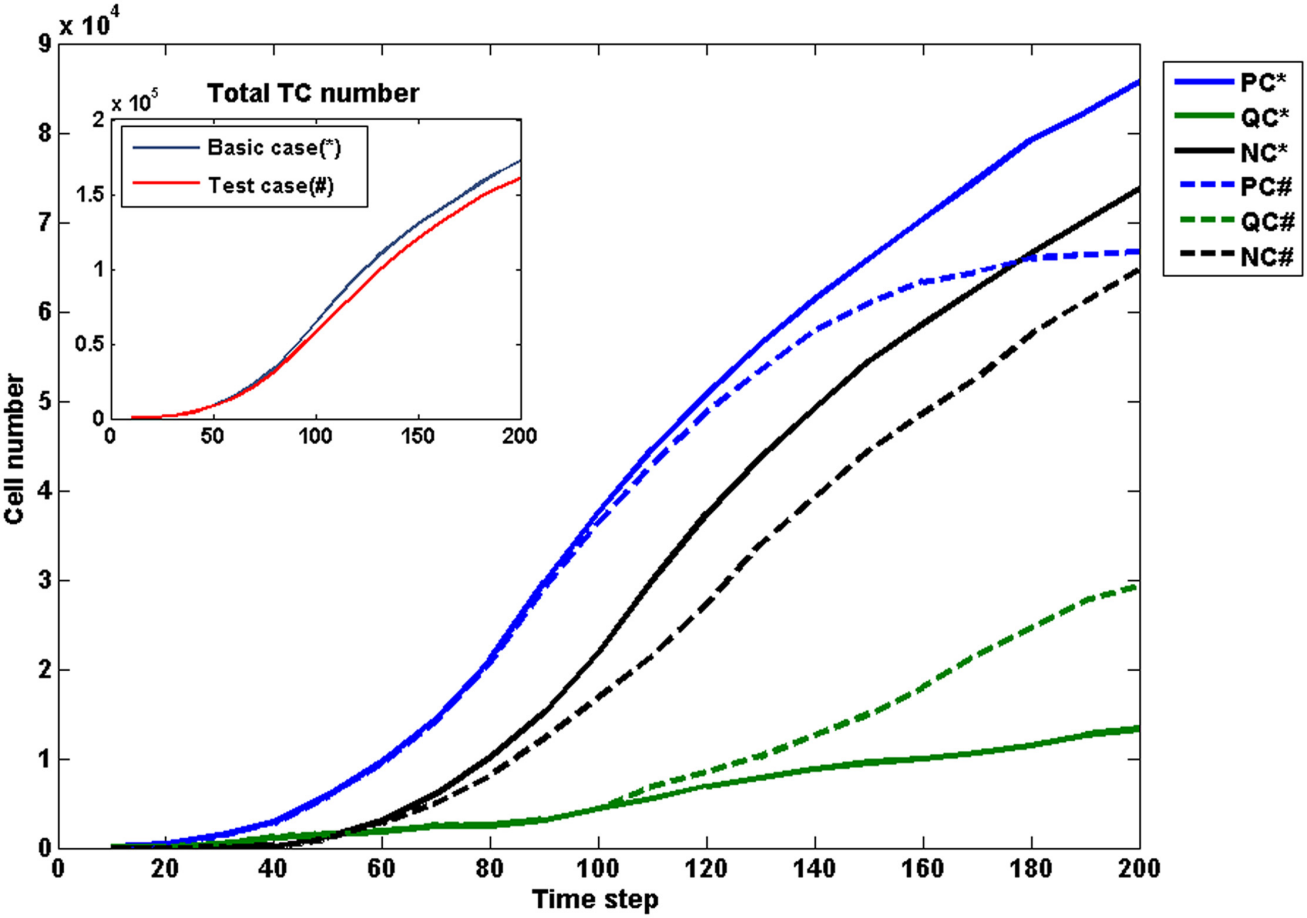
The influence of cell migration on the model. The growth history curves of cell number with different phenotypes and total TC number (inserted panel) in the basic case and test case (C_a). Solid lines: the basic case; Broken lines: the test case C_a.

The phenotype of migrating cells (MC) was excluded in the test model (C_a). As shown in Fig 12, the total number of tumour cells in C_a has a slight reduction compared with the basic case at the end of simulation (T = 200). Significantly, the number of proliferating cells in C_a (blue broken line) reaches a plateau after T = 120. At the same time, the quiescent cells rapidly increase in number (green broken line). However, the number of necrotic cells decreases in C_a (black broken line). Due to the absence of cells migration, the invasion area of tumours in C_a is smaller than that in the basic case. Most cells will gradually become quiescent and necrotic as time goes on. In contrast, the tumour cells migrate along the pre-existing vessels in basic model, leaving more spaces for proliferating cells and quiescent cells, which is one of the factors that result in the formation of hypercellular region on the boundary of pseudopalisade.

#### Vessel remodelling

**Fig 13:**
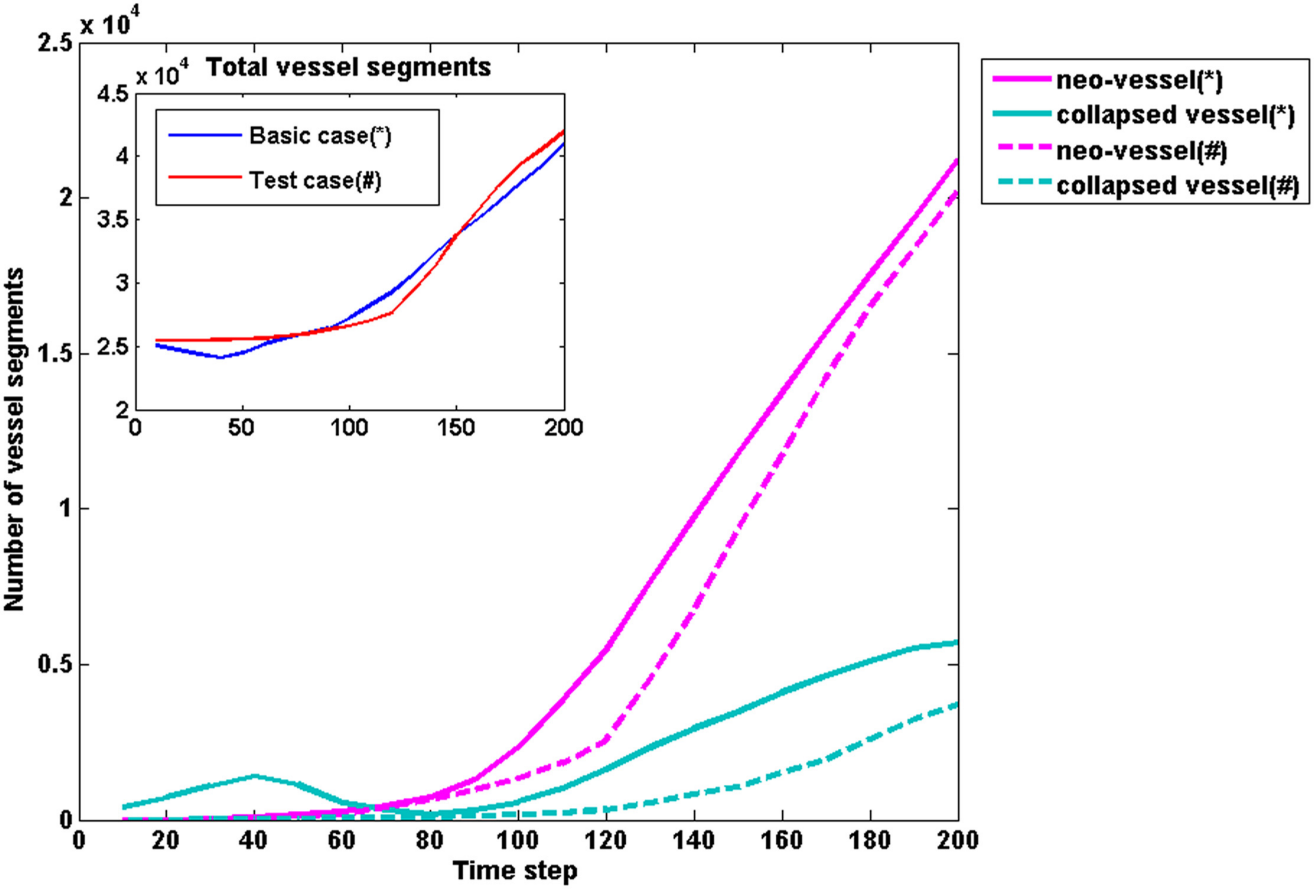
The influence of vessel remodelling on the model. The growth history of vessel segments number in the basic case and test case (C_b). Solid lines: the basic case; Broken lines: the test case C_b.

In the current model, vessel dilation is the first sign of vessel remodelling, leading to a cascade process. Since the key parameters of this remodelling are initiated by the changing in Lp and Pc, the constant Lp and Pc were used in the test model (case C_b), which means the feedbacks between R, Lp and Pc are neglected. The growth history of the number of vessel segments is shown in Fig 13 where case C_b results are presented in broken lines (red line in the inserted panel). Although there is no significant difference in the total number of vessel segments between the two cases (Fig 13, inserted panel), the number of collapsed vessels in C_b is significantly less than that in the basic case. Especially, the sudden increase of collapsed vessels around T = 40 in the basic case does not occur in C_b. This suggests that vessel remodelling caused by the varied Lp and Pc apparently has an effect on the vessel co-option at the early stage. The number of neo-vessels is fewer in case C_b, and the acceleration of neo-vessels occurs later comparing with the basic case. This indicates that the vessel co-option and remodelling at the early tumour development encourages an earlier occurrence of angiogenesis. These combined features in C_b (more functional vessels in the core region and fewer neo-vessels on the boundary region of the tumour) will discourage pseudopalisade formation in a brain tumour case.

#### Flow-dependent oxygen transport

**Fig 14:**
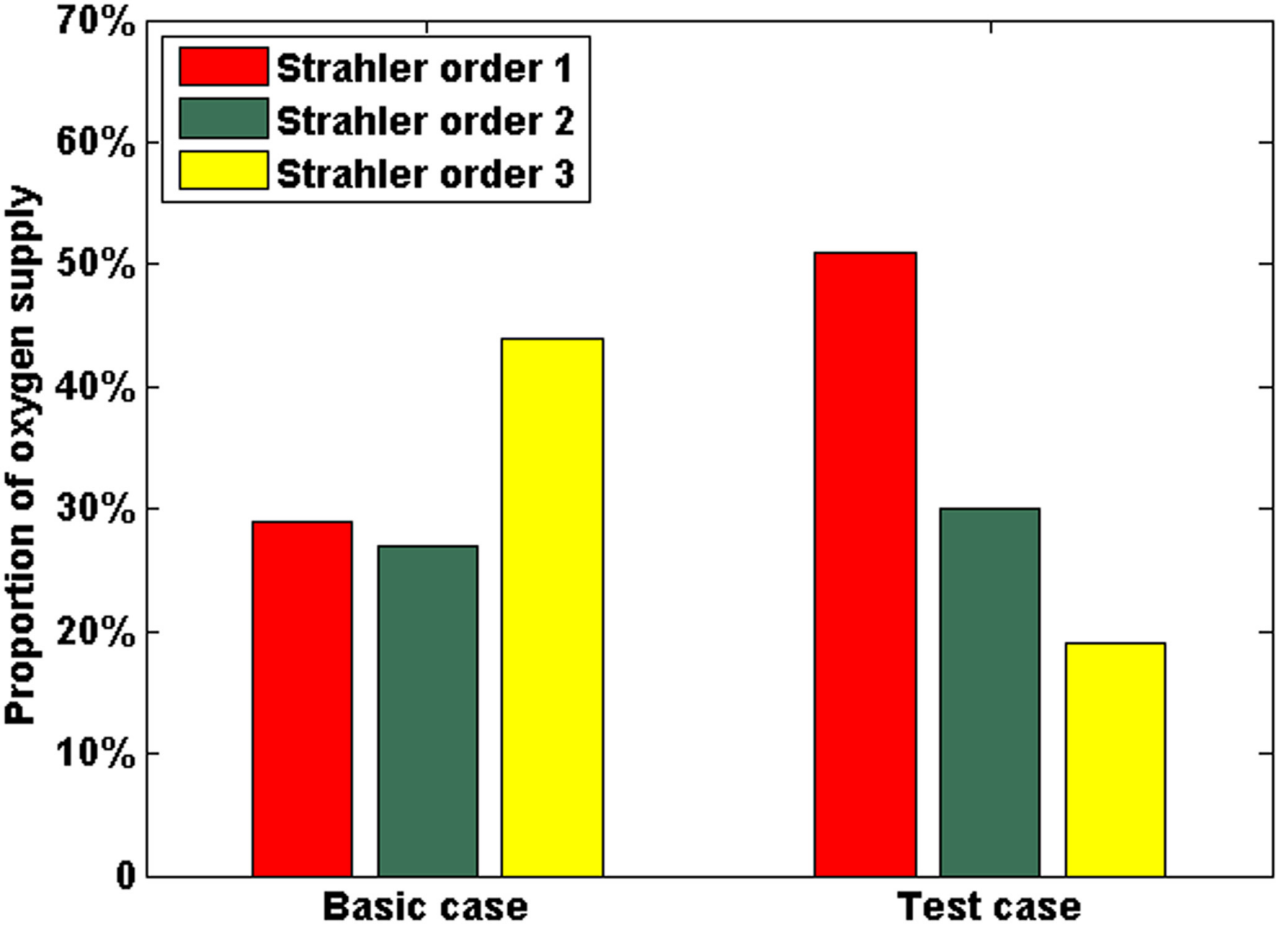
The influence of flow-dependent oxygen transport on the model. The proportion of oxygen supply to the tumour tissue by every Strahler order in the basic case and the test case (C_c) at T = 200.

In the present study, flow-dependent oxygen transport was used instead of the simple treatment of oxygen in our previous studies and many others in which the vessel was treated as point source of oxygen. A test case (C_c) was designed in which the vessel segment was set as a point source of oxygen. The statistical results in Fig 14 show the proportion of oxygen supply to the tumour tissue by every Strahler order at the end of simulation (T = 200). Since the point source of oxygen is only related to the quantity of vessel segments in case C_c, the smallest capillaries (Strahler order 1) with largest number provide the highest percentage of oxygen. In the basic case, although the number of larger vessels (Strahler order 3) is the smallest in the three different sized vessels, the abundant blood perfusion allows them to be the primary provider of oxygen, but at limited perfusion locations. There is no noticeable difference between the two cases in the final results of tumour growth. However, flow-dependent oxygen transport can offer more realistic oxygen concentration field since the blood perfusion is known to be heterogeneous in the tumour tissue.

## Discussion

Based on the coupled model of tumour angiogenesis, tumour growth and blood perfusion in our previous work [30], this study added more coupling features which focus on the early stage of GBM growth. The main improvements compared with the general tumour modelling can be summarized as follows: (a) migrating cells were defined as a new phenotype of GBM cells, (b) a typical tree-like architecture network was generated as an initial vessel network according to the rules of the Strahler system which provided three different sized original vessels, (c) immature vessels undergo remodelling with dynamic variety of vessel radius R and vessel wall permeability Lp in response to the haemodynamic environment, (d) vessel collapse was determined not only by the local wall shear stress, but also by the balance status between vessel dilation and vessel regression. We believe these new assumptions in model setting are responsible for the features which distinguish GBM growth from general solid tumours.

### Cell migration and dysfunctional vessel collapse result in the pseudopalisade formation

In GBM, hypercellular zones typically surround necrotic foci, a specific feature also called pseudopalisades. Two potential mechanisms of pseudopalisade formation presented by Brat *et al* [4] are (a) tumour cells far away from arterial supplies become hypoxic and migrate toward peripheral vessels, leaving a central necrotic zone; (b) vessel occlusion or collapse inside the tumour leads to central hypoxia, followed by tumour cell migration toward a viable blood supply.

In the current model, we defined a phenotype of glioma cells (migrating cells) which can migrate to represent the actively migrating cell population of proliferating cells. Based on the published experimental results [4], it was assumed that the migrating cells have the highest oxygen consumption, VEGF and MDE production. The central tumour region always remains hypoxic, resulting in necrotic zones due to the increased metabolic demands with tumour growth. The migrating cells moved outwardly to the surrounding microvasculature to obtain more oxygen in order to survive, which would result in a population of migrating cells around a central zone (Fig 7A'). This phenomenon will disappear when cell migration ability is removed from the model (Case C_a). This pseudopalisade development result confirms the first mechanism of pseudopalisade formation proposed by Brat *et al* [4].

Based on the current understanding of pseudopalisade formation, not only is the cell migration important, the loss of microvessels in the central region also plays an important role. In the present study, the dynamic of microvasculature involves vessel co-option, remodelling, collapse and angiogenesis depending on the local haemodynamic and chemical environment. Comparing the results shown in Figs 7 and 9, the pseudopalisades gradually invaded new territories by surrounding the capillaries at the tumour peripheral region with the GBM growth. These vessels become structurally and functionally abnormal due to the varied microenvironment caused by the pseudopalisades invasion. As a consequence, some dysfunctional vessels undergo collapse, which leads to local hypoxia. This result demonstrates the second mechanism of pseudopalisade formation. In addition, the number of collapsed vessel segments in the pseudopalisades will increase with the GBM growth in our simulations, which is consistent with the experimental observation [4]. Simulations with cut-off feedback of vessel remodelling (case C_b) also demonstrated the importance of the dysfunctional vessel collapse to the pseudopalisade formation.

### The dynamic remodelling of immature vessels results in the dynamic changing of microvasculature and haemodynamic in GBM

Hyperdilated and hyperplasia blood vessels are characteristics of high-grade brain tumours. There are many experimental studies of metastasis and gliomas indicating that initial GBM growth occurs around co-opted pre-existing blood vessels [6,55,56]. During the co-optive growth, the glioma cells aggregate and migrate along the host vessels longitudinally, resulting in the hypoxia-induced VEGF expression and angiogenesis. In addition, the microenvironment around host vessels also changed due to the production of certain chemical substances (VEGF and ECM concentrations) by tumour cells, which leads to the remodelling of the microvascular network. For vessel remodelling, vessel dilation was assumed to be the first sign of vessel response to the local microenvironment changing, resulting in the increasing of R and Lp. At the same time, an increased Lp will cause the Pc (vessel collapse pressure) to be smaller and the interstitial fluid pressure Pi increases, which can both induce vessel compression, *i*.*e*., vessel diameter reduction. In the simulation, the microvasculature maintains an equilibrium state with dynamic vessel remodelling, resulting in the dynamic changing of vascular network and haemodynamics (Fig 9). At the same time, a changed microvasculature will influence the GBM growth. This coupled dynamic progress makes the model more realistic.

A low WSS for a period of time will cause a micro vessel collapse. However, the defining of this WSS threshold value can be a problem. Simulations were carried out by changing the critical collapse WSS value *τ*_*crit*_ from 0.2f_0_ to 0.8f_0_ to analyse the vessel segment number changing against the total number of tumour cells. Results shown that a *τ*_*crit*_ = 0.5f_0_ will produce a more stable vessel number and TC number relationship.

### The location of initial tumour cells and surrounding microenvironment result in the GBM growth curve

The influence of the initial tumour cell location on the tumour growth is significant (Fig 10). It can be seen that although migration allows tumour cells to move to other sites to grow, the size and the growth speed of the tumour is significantly smaller and slower than the case in which the initial tumour cells are planted in the well perfused region. The feature agrees very well with what was observed by Zhao *et al* in their experimental study [9]. We can deduce from this result that the co-option of pre-existing vessels is an alternative choice for GBM cells to survive in a blood-supply-deficient microenvironment until they migrate to a blood-supply-sufficient microenvironment to initiate exponential growth through angiogenesis. The role of cell migration along host vessels is noteworthy in the GBM pathological research and anti-angiogenic therapies.

The simulation of the current model focuses on the avascular phase of tumour development and stopped at an early phase of angiogenesis. The model is able to demonstrate the main features of GMB growth in this phase such as the formation of pseudopalisades, the influence of initial conditions and the environment local to the early phase tumour growth. Despite the great efforts on making the model more realistic, there are a few major limitations of the work: (1) On calculating the tumour mechanical environment, only interstitial fluid static pressure was included. Mechanical stress caused by the rapid proliferation of tumour cells was not simulated in the model. This solid mechanical stress may influence tumour cell behaviour; (2) Although most of the simulation parameters were set based on published experimental data, some of them cannot be found, such as tumour migration speed. One grid space per time step was used in the study which does not have direct experimental support. The real migration speed can be fast or slower than this. As a consequence, the total migration distance can be artificial. This part of the result can only be interpreted as qualitative rather than quantitative.

## Conclusion

In this work, we have proposed a dynamic mathematical modelling system to investigate the early growth process of glioblastoma by coupling the chemical and haemodynamic microenvironment caused by pre-existing vessel co-option, remodelling, collapse and angiogenesis. A 3D tree-like architecture network with different orders for vessel diameter is generated as pre-existing vasculature in host tissue. The model confirms the two different mechanisms of pseudopalisade formation by study of the dynamic relationships between the pseudopalisades, migrating cell distribution and the co-option and remodelling of pre-existing vessels. The model is not only able to provide the global results given above, but also to investigate the local immature vessel remodelling such as co-option, dilation, leaky, angiogenesis, regression and collapse and its influence on the local microenvironment and GBM growth.

We studied the influence of initial glioma cell planting location on the GBM growth. Although the glioma cells were located in a blood-supply-deficient microenvironment initially, the ability of cell migration allowed them to move along pre-existing vessels and survive, until they migrated to a blood-supply-sufficient microenvironment to initiate exponential growth through angiogenesis. This suggests that the role of cell migration along host vessels is noteworthy in the GBM pathological research and anti-angiogenic therapies. Furthermore, the influences of newly added feedbacks including cell migration and vessel remodelling on the proposed model were discussed. We believe these new assumptions in model setting are responsible for the features which distinguish GBM growth from general solid tumours.
